# Effects of sun angle, lunar illumination, and diurnal temperature on temporal movement rates of sympatric ocelots and bobcats in South Texas

**DOI:** 10.1371/journal.pone.0231732

**Published:** 2020-04-23

**Authors:** John P. Leonard, Michael E. Tewes, Jason V. Lombardi, David W. Wester, Tyler A. Campbell

**Affiliations:** 1 Caesar Kleberg Wildlife Research Institute, Texas A&M University-Kingsville, Kingsville, Texas, United States of America; 2 Natural Heritage New Mexico, Museum of Southwestern Biology, University of New Mexico, Albuquerque, New Mexico, United States of America; 3 East Foundation, San Antonio, Texas, United States of America; U.S. Geological Survey, UNITED STATES

## Abstract

Sympatric ocelots (*Leopardus pardalis*) and bobcats (*Lynx rufus*) in South Texas show substantial overlap in body size, food habits, and habitat use. Consequently, we explore whether temporal niche partitioning may explain ocelot and bobcat coexistence. We investigated the influence of sun angle, lunar illumination, and maximum diurnal temperature on temporal movement rates of sympatric ocelots (n = 8) and bobcats (n = 6) using a combination of high-frequency GPS locations and bi-axial accelerometer data. We demonstrated that accelerometer data could be used to predict movement rates, providing a nearly continuous measure of animal activity and supplementing GPS locations. Ocelots showed a strong nocturnal activity pattern with the highest movement rates at night whereas bobcats showed a crepuscular activity pattern with the highest movement rates occurring around sunrise and sunset. Although bobcat activity levels were lower during the day, bobcat diurnal activity was higher than ocelot diurnal activity. During warmer months, bobcats were more active on nights with high levels of lunar illumination. In contrast, ocelots showed the highest nocturnal activity levels during periods of low lunar illumination. Ocelots showed reduced diurnal activity on hotter days. Our results indicate that ocelot and bobcat coexistence in South Texas can be partially explained by temporal niche partitioning, although both felids showed periods of overlapping activity during nocturnal and crepuscular periods.

## Introduction

The ocelot (*Leopardus pardalis*) is a federally endangered species in the United States [1]. Its range has constricted since the 1800s primarily due to anthropogenic land conversion of native habitat [2]. In the United States, the ocelot is now confined to two isolated populations in South Texas, one on the Laguna Atascosa National Wildlife Refuge (LANWR) and the other on private ranches in Willacy and Kenedy counties, TX [3, 4]. In Central and South America, ocelots have been recorded in mangrove (*Rhizophora spp*.) forests, coastal marshes, savanna grasslands, thorn scrub, tropical forest, and subtropical forest [5–7]. However, in South Texas the ocelot is primarily associated with evergreen forests dominated by live oak (*Quercus* spp.) and dense thornshrub forests [8–16].

Throughout much of its geographic range, the ocelot is sympatric with 6 other felids: bobcat (*Lynx rufus*), jaguar (*Panthera onca*), puma (*Puma concolor*), jaguarundi (*Puma yagouaroundi*), margay (*Leopardus wiedii*), and oncilla (*Leopardus tigrinus*) [17]. Coexistence of ocelots with these other species can largely be explained by differences in morphology [18], habitat selection, and diel activity patterns [19,20]. The ocelot is several times smaller than the jaguar and puma, and 2 to 4 times larger than the jaguarundi, margay, and oncilla [7, 21], reducing direct competition with ocelots for prey items.

In contrast to the ocelot, the bobcat is the most widely distributed and abundant wild felid in North America, with populations believed to be increasing [22]. Ocelots and bobcats show substantial body size overlap. Male bobcats typically average 10 kg, whereas females average 7 kg, although these weights vary with latitude [6, 23,24]. Adult ocelots in South Texas typically weigh between 7 and 10 kg [9]. Bobcats occur in a variety of habitats in the United States, including boreal and coniferous forest, bottomland hardwood forest, coastal swamp, desert, urban areas, and scrubland [25,26]. In Mexico, bobcats use pine-oak woodlands, dry scrub, grassland, and tropical dry forest [27,28]. Although bobcats can occupy a wider variety of habitats than ocelots, in Texas they are closely associated with the same dense thornshrub communities used by ocelots [29,30]. At the home range level, Horne et al. [15] found few significant differences in the habitat used by ocelots and bobcats within LANWR, although evidence of spatial niche partitioning was observed at a micro-habitat scale.

Ocelots primarily feed on small mammals (e.g., *Neotoma* spp., *Peromyscus* spp., *Lyomys* spp., *Reithrodontomys* spp., *Baiomys* spp., *Sigmodon* spp., *Agouti* spp., and *Sylvilagus* spp.), although they have also been reported to eat young white-tailed deer (*Odocoileus virginianus*), small reptiles, birds, and fish [5]. Although bobcats consume prey as large as adult white-tailed deer [31,32], they feed primarily on small mammals, similar in size to prey preferred by ocelots [33–36]. In South Texas, scat analyses revealed high dietary overlap between ocelots and bobcats [37], indicating that the two species may compete for the same prey.

According to the competitive-exclusion theory [38], ecologically equivalent species cannot coexist. Ecological similarities in habitat use, diet, and body size suggest that sympatric ocelots and bobcats will show temporal niche partitioning and focus activity during different diel periods. Although bobcats are commonly described as nocturnal with crepuscular activity peaks, bobcat eyes are proportionally smaller than those of strictly nocturnal cats [23, 39], allowing bobcats to hunt diurnally and nocturnally [7, 24, 40–42]. Ocelots have also been described as nocturnal and crepuscular, with activity peaks occurring at dawn and dusk [5, 7, 43, 44].

Previous studies of ocelot activity patterns have primarily relied on camera trapping [e.g., 19, 45, 46]. Investigations of animal activity patterns using camera traps are based on the assumption that the frequency of photographic captures obtained during a time interval will be proportional to animal activity levels during that time interval [47]. Camera trapping is a cost-effective way to estimate gross activity patterns for elusive animals such as ocelots. However, the number of photographic captures obtained through camera trapping may be relatively low in a given time period for animals with elusive behaviors and low population densities such as ocelots. Low capture rates may affect the reliability of subsequent activity curve estimates, and may preclude the examination of interacting factors such as temperature, season, and moon phase [47].

Radio and GPS telemetry enables researchers to observe animal activity patterns at the individual level. Modern GPS collars allow for the collection of GPS fixes throughout different diel periods, seasons, and moon phases. Linear distance between consecutive GPS fixes can be divided by the corresponding time lag to estimate velocity, and these velocity values may be used as a surrogate for overall activity [48]. High-frequency track schedules entail a reduction in battery life, and researchers must balance between track schedule intensity and duration to fulfill stated objectives. In contrast, accelerometers provide continuous monitoring of animal behavior with extremely low reduction in battery life [49]. Accelerometers have been used to study foraging, reproduction, activity, energy budgets, and locomotion [49–51]. When used in conjunction with GPS locations, accelerometers can be a powerful tool for studying animal movement and activity patterns.

The objective of this study was to investigate the effects of lunar illumination, sun angle, and maximum diurnal temperature on movement and activity patterns of sympatric ocelots and bobcats using a combination of high-frequency GPS telemetry data and continuously collected accelerometer data. An additional objective of this study was to determine whether accelerometer data could be used to accurately predict movement rates for ocelots and bobcats. To compare ocelot and bobcat temporal activity patterns we developed the following *a-priori* hypotheses: 1) Ocelots would display a typical nocturnal activity pattern with high activity levels at night, intermediate activity levels during the crepuscular period, and low activity levels during the day, 2) Bobcats would show a crepuscular movement pattern, with high peaks of activity around dawn and dusk, varying levels of activity at night, and occasional bouts of activity during the day, 3) Nocturnal activity levels for bobcats would be highest during periods of high lunar illumination, 4) Nocturnal activity levels for ocelots would be highest during periods of low or intermediate lunar illumination, and 5) Both ocelots and bobcats would reduce diurnal activity levels during the hotter time of year to avoid thermal stress.

## Materials and methods

### Study area

The study was conducted on the East Foundation’s El Sauz Ranch, a 113 km^2^ ranch owned and managed by the East Foundation near Port Mansfield, Texas (26.54° N, 97.44° W). Climate of the El Sauz Ranch is typical of the Coastal Sand Plain, Lower Rio Grande Valley, and Laguna Madre Barrier Islands and Coastal Marshes eco-regions, with mean temperatures ranging from 16°C to 28°C, a mean annual precipitation of 68 cm, and substantial inter-annual variation in rainfall patterns [52,53]. Major land cover types include dense live oak (*Quercus virginiana*.) forests and mottes (groves or stands of trees surrounded by open-canopy environments), emergent palustrine wetlands, open grassland, and thornshrub. Common woody plants found associated with ocelot habitat include spiny hackberry (*Celtis pallida*), crucita (*Eupatorium odoratum*), Berlandier fiddlewood (*Citharexylum berlandieri*), honey mesquite (*Prosopis glandulosa*), desert olive (*Forestiera angustifolia*), snake-eyes (*Phaulothamnus spinescens*), colima (*Zanthoxylum fagara*), and brasil (*Condalia hookeri*) [54].

### Capture and telemetry

From 2013 to 2017, we trapped ocelots and bobcats with single-door 108 x 55 x 40 cm wire box traps (Tomahawk Trap Co., Tomahawk, WI), baited with live chickens or pigeons, maintained safely in a separate enclosure. Trapped ocelots and bobcats were sedated with a mixture of tiletamine HCL and zolazepam (Telazol, Fort Dodge Laboratories, Fort Dodge, Iowa), at a dosage of 5 mg per kg body weight [55]. Animal weight was visually estimated, and drugs were administered with a pole syringe. Animal handling was conducted in compliance with, and approved by, the Texas A&M University-Kingsville Institutional Animal Care and Use Committee protocol numbers 2012-12-20B-A2, 2012-12-20B, 2012-12-19, and 2015-12-21B-A4; United States Fish and Wildlife Service permit number PRT-676811; and Texas Parks and Wildlife Department permit number SP0190-600.

Animals were fitted with Minitrack or Litetrack GPS collars manufactured by Lotek (Lotek Wireless, New Market, Ontario, Canada) or G2110G GPS collars manufactured by ATS (Advanced Telemetry Systems, Insanti, MN, USA). The ATS collars were fitted to bobcats captured in 2013, and were programmed according to the following track schedule: one location each 24-hours at noon (1200 hr.), a second location at midnight (0000 hr.), and a high-frequency track period with locations recorded every 30 minutes for a 72-hr. period centered around each full moon and each new moon night. Lotek Minitrack GPS collars were used from 2014 to 2017. To optimize battery life, we reduced the 72-hr. high-frequency monitoring to a 24-hr. schedule centered on each full moon and each new moon night, but otherwise programmed the collars according to the same schedule as the ATS collars. In 2017, we also deployed Lotek Litetrack 130 GPS Collars, programmed to record locations continuously every 30 minutes throughout the entire study period.

### Movement data

We calculated movement distance between consecutive high-frequency GPS fixes (~ 30 min interval) and removed GPS fixes that were not collected at high frequency or that had horizontal dilution of precision (HDOP) >10 [56]. We calculated straight-line distance (m) between consecutive GPS fixes and divided this by the corresponding time lag (hr.) to obtain estimated movement velocity.

### Accelerometer data

In addition to recording GPS fixes, the Lotek Minitrack collars contained bi-axial accelerometers which measured activity 4 times each second simultaneously on the horizontal (ActivityX) and vertical (ActivityY) axes throughout the entire period the animal was collared. Activity values were reported on each axis as the difference in acceleration between 2 consecutive measurements. Values were recorded between 0 and 255 and reported at 5 minute intervals. Each individual collared with a Lotek Minitrack collar, therefore, had two independent measures of movement: GPS fixes, which were recorded at high frequencies (i.e., 30 min) only during full moon and new moon periods, and accelerometer data, which were reported continuously at 5-min intervals.

### Predicting movement velocity from accelerometer data

A preliminary comparison of velocity and accelerometer values (ActivityX and ActivityY) in animals collared with Lotek GPS collars revealed a positive correlation between these variables, suggesting that accelerometer data could be used to predict velocity. Because high-frequency GPS fixes were recorded at 30-min intervals and each accelerometer reading was reported at 5-min intervals, each GPS-based velocity value corresponded to six accelerometer-based measures each for ActivityX and ActivityY. For each unique pair of consecutive GPS fixes recorded at 30 min intervals, we used the unique timestamp to attribute the ending location with the minimum, maximum, mean, and variance of the 6 preceding ActivityX and ActivityY values. We used the R package randomForest [57, 58] to apply a random forest regression [59], using the summary statistics calculated from activity readings as independent variables and velocity as the dependent variable. We used the default value of *p*/3 for the number of variables tried at each split, where *p* is the number of predictor variables. We set *ntree*, the number of trees grown in the forest, to 1,000. To determine our success in predicting movement velocity from accelerometers we used the random forest model to predict velocity across the training data set. We then performed a simple linear regression between actual and predicted velocity values to determine how closely actual velocity values (i.e., those obtained through GPS fixes) related to predicted velocity values (i.e., those estimated from accelerometer data). We considered the prediction successful if actual and predicted velocity showed a strong, positive linear relationship with a high R^2^ value.

To ensure that the relationship between accelerometer values and velocity was not dependent on species, moon phase, sun angle, or temperature, we additionally created a series of multiple linear regression models that included each of these variables as an interactive term along with predicted velocity. For each multiple linear regression model, we report the percentage of variation explained by the addition of the interactive term. After training the random forest model on actual movement values obtained from high-frequency GPS fixes, we applied the model to the entire set of accelerometer data for each individual. This allowed us to obtain estimated movement rates, based solely on accelerometer data, for the entire time period each individual was tracked. Movement values predicted through random forest regression of accelerometer values were termed “Predicted Velocity”, whereas those obtained through GPS fixes are hereafter termed “True Velocity”.

For all individuals tracked with both high-frequency GPS fixes and accelerometers, we combined Predicted Velocity and True Velocity into a field named “Velocity Combined” using the following rule set: 1) Where available, True Velocity would be considered the best available measure of velocity and would be the value used in the Velocity Combined field, 2) During times where high-frequency GPS data were unavailable, Predicted Velocity would instead be used in the Velocity Combined field as a measure of animal velocity. This approach allowed us to augment the velocity measurements available for each individual, using GPS-based True Velocity measures where available, and using accelerometer-based Predicted Velocity during periods where we lacked high-frequency GPS readings. For individuals with GPS-collars that lacked accelerometers, all values in the Velocity Combined field represent True Velocity readings. To differentiate between True and Predicted Velocity we added the binary field “Type” that indicates, for a particular velocity estimate, whether the value was derived from GPS or accelerometer data.

Our subsequent comparisons between ocelot and bobcat movement rates throughout various diel-based and lunar-based time periods assume similar baseline movement rates between these species. We therefore scaled and centered Velocity Combined separately for ocelots and bobcats. To meet assumptions of normality, we logarithmically transformed these scaled and centered velocity values, naming the transformed variable “log Velocity”, which was used as the dependent variable in all subsequent analyses.

### Weather, moon phase, and sun angle data

**Fig 1:**
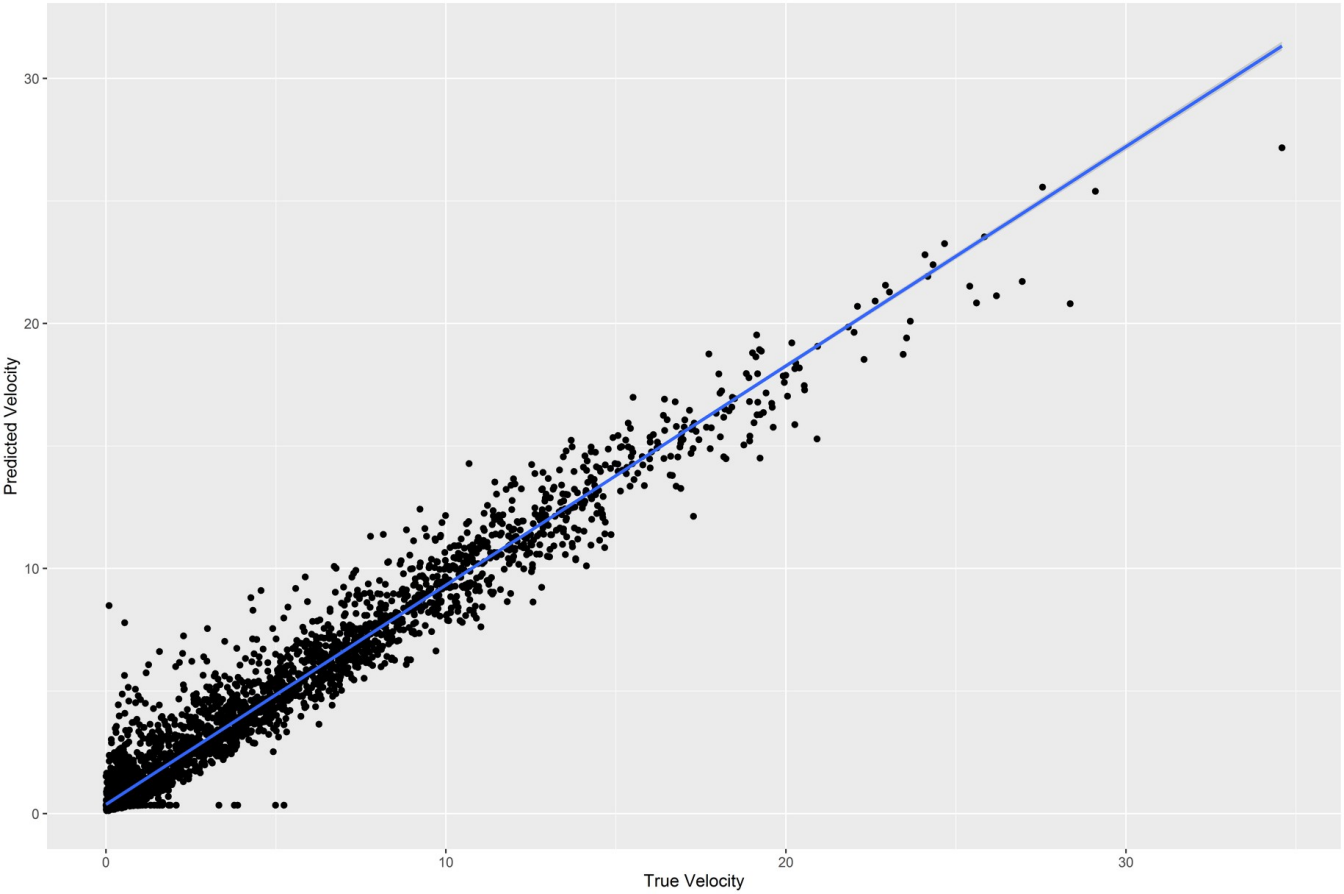
Comparison of true and predicted velocity using random forests regression. Predicted Velocity (m/hr.) was plotted against True Velocity (m/hr.) for ocelots and bobcats, trapped on the East Foundation’s El Sauz Ranch between 2013 and 2017, for which GPS and accelerometer data were collected. The blue trend line shows the ordinary least squares line for the regression of Predicted Velocity on True Velocity.

We used the R package oce [60] to obtain the following location-specific data for each unique timestamp within the GPS and accelerometer datasets: 1) “MoonIlluminatedFrac”- the decimal proportion (0 to 1) of the moon’s surface illuminated, 2) “MoonAlt”- the altitude of the moon, expressed as degrees above (positive) or below (negative) the horizon, and 3) “SunAlt”- the altitude of the sun, expressed as degrees above (positive) or below (negative) the horizon. We downloaded historical weather data from the National Oceanic and Atmospheric Administration (NOAA) for Harlingen International Airport (KHRL), the closest weather observation station. The only temperature-based variables available for the entire study period were TMIN (the minimum temperature for a given 24 hour period) and TMAX (the maximum temperature for a given 24 hour period). We found TMAX and TMIN to be highly correlated with each other (Pearson’s correlation coefficient = 0.85), precluding the inclusion of both variables in a linear model. Our primary interest was in determining how ocelot and bobcat movement patterns differed between periods characterized by hot vs. cool daytime temperatures. As such, the only weather-derived variable included in subsequent analyses was TMAX. To visually explore the overall movement patterns of ocelots and bobcats due to diel period, we partitioned SunAlt into 10° blocks and plotted mean True Velocity and mean Predicted Velocity separately as bar plots for ocelots and bobcats (Fig 1).

### Preliminary mixed-effects models

We constructed a series of additive and interactive linear mixed effects models to explore the relationship between temperature, lunar illumination, and sun angle, using the lme function in the R package nlme [61]. Specifically, we used SunAlt, MoonIlluminatedFrac, and TMAX as independent variables in 9 competing linear mixed effects models with log Velocity as the dependent variable. All independent variables were left as continuous predictors, and were not categorized into discrete factor levels. We fit models independently for ocelots and bobcats. All models included individual ID and Type (i.e., accelerometer vs. GPS) as random effects, and incorporated a first-order autoregressive pattern (AR1) to account for the temporal autocorrelation inherent in the data set [62]. Models were fit using maximum likelihood and ranked according to Akaike information criterion (AIC). We considered the model with the lowest AIC value to represent the best-fitting model for that species [63].

### Defining discrete time periods for activity comparison

We categorized all diel periods into “Day”, “Night” and “Crepuscular” based on the following rule set: times where the sun was > 13.5° above the horizon were defined as Day, times where the sun was > 13.5° below the horizon were defined as Night, and times where the sun was between these values were defined as Crepuscular. We selected these thresholds because the lower limits for SunAlt roughly corresponded to either the beginning of morning civil twilight or to the end of evening civil twilight, common definitions for the start and end of the crepuscular period. The upper range of SunAlt values allowed in the Crepuscular definition generally corresponded to periods no later than 1 hour after sunrise or 1 hour before sunset, time periods during which cooler temperatures and lower light levels generally prevail.

We categorized moon phase into “Dark Moon”, “Mid Moon”, and “Full Moon” based upon a combination of MoonIlluminatedFrac and MoonAlt (i.e., angle of the moon above or below the horizon). Full Moon periods were defined as those times when MoonIlluminatedFrac was ≥ 0.9 and MoonAlt was > 0 (i.e., the moon was above the horizon). Dark Moon periods were defined as those times when MoonIlluminatedFrac was < 0.1 or MoonAlt was < 0 (i.e., moon was below the horizon). This allowed us to identify all time periods where lunar illumination was low or absent, whether this was the result of moon phase or of the moon being below the horizon. All time periods where MoonIlluminatedFrac was ≥ 0.1 but <0.9 and Moon Angle was > 0 were termed Mid Moon. This category accounted for periods with intermediate lunar illumination. Our designations of “Dark Moon” and “Full Moon” coincide with the thresholds used by previous studies (e.g., Rockhill et al. [48]), allowing comparability between studies.

Our interest in including temperature in the analysis was specifically to test the hypothesis that ocelots and bobcats would shift to a more nocturnal activity pattern during periods with high diurnal temperatures to avoid thermal stress. As such, we used the concept of thermoneutrality to set our threshold for defining “Hot” vs. “Cool” time periods. The thermoneutral zone is defined as the range of temperatures over which an animal’s metabolism is relatively constant and not influenced by temperature [64]. Animals are expected to experience thermal stress at temperatures above and below the thermoneutral zone, with changes in temporal activity patterns a potential behavioral adaptation to high temperatures [65]. McNab [66] reported the zone of thermoneutrality to be from 22 to 33 °C for ocelots, 19 to 32 °C for smaller bobcats, and 13 to 30 °C for larger bobcats. To identify dates where both ocelots and bobcats would likely experience daytime thermal stress, we categorized all dates with a regional maximum temperature (TMAX) ≥ 33 °C as “Hot” and all other dates as “Cool”, naming the resultant variable “Season”.

We believed that lunar illumination levels would only be likely to influence ocelot and bobcat movement rates during the nocturnal and crepuscular periods. Simply including moon phase and diel period as interactive effects would complicate the analysis by introducing factor levels that we felt would be uninformative (e.g., “Full Moon Day”). As such, we created a new categorical variable termed “LunarDiel” which included the interaction between moon phase and diel period only for Night and Crepuscular periods. The factor levels for this variable were “Dark Moon Night”, “Mid Moon Night”, “Full Moon Night”, “Dark Moon Crepuscular”, “Mid Moon Crepuscular”, “Full Moon Crepuscular”, and “Day”.

Our final model included LunarDiel, Season, and Species as interactive terms in a linear mixed effects model. Individual ID and Type (i.e., Predicted vs. True Velocity) were included as random effects, and the model incorporated a first-order autoregressive structure (AR1) to account for the serial autocorrelation inherent in the data set [62]. We conducted *post-hoc* comparisons between combinations of Species, LunarDiel, and Season using estimated marginal means in the R package emmeans [67]. Estimated marginal means allow pairwise comparisons between groups using a reference grid consisting of combinations of factor levels, with each covariate set to its mean value. For each period, we computed a 95% confidence interval for log Velocity values independently for ocelots and bobcats. Additionally, we performed pairwise comparisons of estimated marginal means between all factor levels using Tukey-adjusted comparisons, setting alpha to 0.05. We performed two distinct types of comparisons using emmeans: 1) comparisons of activity between time periods for a given species, and 2) comparisons of ocelot and bobcat activity within a given time period. This allowed us to test hypotheses related to each species’ temporal activity patterns as well as compare predicted activity levels between the two species to identify periods of temporal niche overlap.

## Results

### Capture and telemetry

Between 2013 and 2017 we captured and collared 8 ocelots (4M, 4F) and 6 bobcats (5M, 1F). We deployed ATS GPS collars on 2 bobcats (1M, 1F), Lotek Minitrack GPS collars on 6 ocelots (2M, 4F) and 4 bobcats (4M), and Lotek Litetrack GPS collars on 2 ocelots (2M). Only the Lotek Minitrack GPS collars had accelerometers which could be used to generate Predicted Velocity values. After censoring GPS datasets to include only high-frequency locations with HDOP <10 [56], our GPS dataset consisted of 11,621 measures of True Velocity. Among individuals collared with Lotek Minitrack collars, we recorded 354,851 accelerometer readings (Table 1).

**Table 1:** Track period, GPS location schedule, number of GPS locations, and number of accelerometer values obtained for ocelots and bobcats captured on the East Foundation’s El Sauz Ranch.

ID	Species	GPS Locations	Track Period	GPS Schedule	Accelerometer Values
Y12M^1^	Ocelot	525	16 Mar 2014–9 Sep 2014	24-hr full/new moon	41,725
E6M^1^	Ocelot	747	20 Apr 2015–25 Jan 2016	24-hr full/new moon	80,452
E15M^2^	Ocelot	220	14 Jan 2017–1 Feb 2017	Continuous	NA
Y5M^2^	Ocelot	1171	16 Jan 2017–27 Mar 2017	Continuous	NA
E10F^1^	Ocelot	333	1 Mar 2014–13 Jul 2014	24-hr full/new moon	41,784
E12F^1^	Ocelot	545	20 Mar 2015–29 Sep 2015	24-hr full/new moon	53,748
E17F^1^	Ocelot	2841	25 Jan 2017–27 Mar 2017	Continuous	2,975
E14F^1^	Ocelot	269	22 Apr 2016–20 Sep 2016	24-hr full/new moon	30,447
EB16M^3^	Bobcat	1553	9 May 2013–10 Jul 2013	72-hr full new moon	NA
EB8M^1^	Bobcat	438	4 Apr 2015–15 Aug 2015	24-hr full/new moon	41,816
EB18M^1^	Bobcat	369	7 Apr 2016–20 Jul 2016	24-hr full/new moon	38,390
EB21M^1^	Bobcat	43	24 Feb 2017–25 Feb 2017	Continuous	17,568
EB20M^1^	Bobcat	886	5 Mar 2017–24 Mar 2017	Continuous	5,946
EB15F^3^	Bobcat	1681	26 Apr 2013–20 Oct 2013	72-hr full new moon	NA

Summary of ocelots and bobcats captured and collared with GPS collars between 2013 and 2017. The individual identifier is given by the field “ID”. Males and females are indicated by a suffix of “M” or “F”, respectively under the ID field. GPS Schedule indicates the period during which GPS fixes were recorded at 30 min intervals.

^1^Individual collared with Lotek Minitrack GPS Collar.

^2^Individual collared with Lotek Litetrack 130 GPS Collar.

^3^Individual collared with ATS GPS Collar.

### Predicting movement velocity from accelerometer data

An exploratory comparison between GPS-derived velocity and original accelerometer values revealed a strong positive relationship between velocity and corresponding ActivityX and ActivityY values, with higher accelerometer values indicative of greater movement rates. However, two individuals (1 male bobcat, 1 female ocelot) showed several high-velocity movements during which accelerometer-based readings were abnormally low. We considered this pattern related to accelerometer malfunction and censored these individuals from the movement prediction analysis. After removing these individuals and censoring GPS values not recorded during the high-frequency track period, we had 3,984 GPS readings that we were able to attribute with corresponding accelerometer values. Contemporaneously recorded ActivityX and ActivityY values were highly correlated (Spearman’s correlation coefficient = 0.96) indicating that they could not be treated as independent measures of activity. Additionally, accelerometer values showed substantial temporal autocorrelation, approximating a first-order autoregressive pattern.

For the random forest prediction of velocity, the simple linear regression performed between Predicted Velocity and True Velocity showed a strong positive relationship between the two variables (R^2^ = 0.9597, adjusted R^2^ = 0.9597, F-statistic = 9.474e+04 on 1 and 3982 DF, p-value <2.2e-16). The random forests model explained 72.88% of the variance in True Velocity, and had a Root Mean Squared Error of 6.18. Using a studentized Breusch-Pagan test, we rejected the null hypothesis of homoscedasticity (BP = 131.51, df = 1, p-value p 2.2e-16) and assumed a heterogeneous variance of the residuals. Examination of a plot between True Velocity and Predicted Velocity showed variance of the residuals to increase slightly at higher velocity values (Fig 1). For the multiple regression models that included interactions with potentially confounding variables, we found that the interaction between True Velocity and Species, SunAlt, MoonIlluminatedFrac, and TMAX accounted for 0.09%, 0.02%, <0.01%, and 0.02% of the variation in Predicted Velocity, respectively. These results indicate that the relationship between True Velocity and Predicted Velocity does not depend substantially on Species, SunAlt, MoonIlluminatedFrac, or TMAX.

### Diel-based movement patterns of ocelots and bobcats

**Fig 2:**
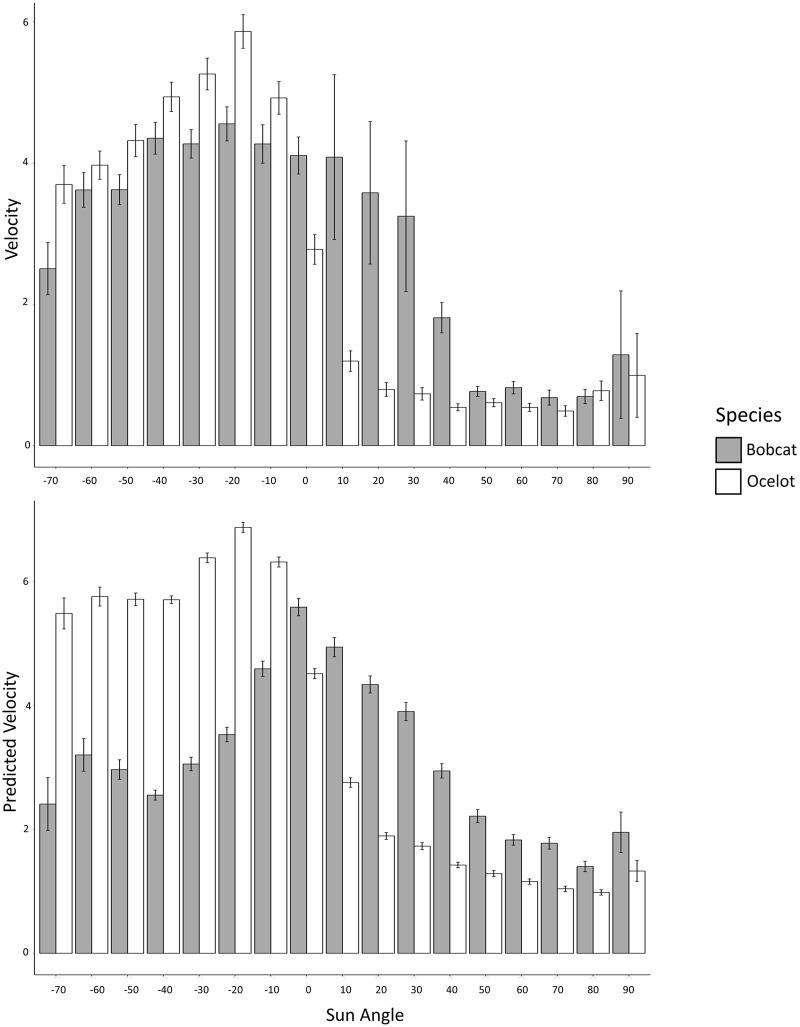
Actual and predicted movement velocity for ocelots and bobcats. Velocity (m/hr.) values are averaged over 10° increments in Sun Angle. The top figure displays GPS-derived velocity values for ocelots and bobcats trapped on the East Foundation’s El Sauz Ranch between 2013 and 2017. The bottom figure displays accelerometer-derived Predicted Velocity values for ocelots and bobcats that were collared with Lotek Minitrack collars. Negative values of Sun Angle refer to degrees below the horizon and positive values refer to degrees above the horizon. Error bars represent standard error of the mean.

A visual comparison of ocelot and bobcat velocity by Sun Angle revealed ocelots to have higher movement rates during times with negative Sun Angle values (i.e., night) relative to bobcats. In contrast, bobcats appeared to be more active during times with positive Sun Angle values (i.e., day) relative to ocelots. Using the Predicted Velocity dataset, this pattern was more pronounced, with Predicted Velocity values much higher for ocelots than bobcats at night and higher for bobcats than ocelots during the day (Fig 2).

### Exploratory analysis of temperature, lunar illumination, and sun angle

For both ocelots and bobcats, Model 9, the global model consisting of SunAlt, MoonIlluminatedFrac, and TMAX, plus interaction terms, was selected as the top model (Table 2). An examination of Model 9 coefficients for ocelots and bobcats revealed significant fixed effects for bobcats for the following covariates: SunAlt, MoonIlluminatedFrac, TMAX, SunAlt:TMAX, and MoonIlluminatedFrac:TMAX. For ocelots, there were significant fixed effects for the following covariates: SunAlt, TMAX, SunAlt:MoonIlluminatedFrac, SunAlt:TMAX, and SunAlt:MoonIlluminatedFrac:TMAX (Table 3).

**Table 2:** Comparison of 9 additive and interactive linear mixed-effects models fit independently for ocelots and bobcats.

Model	Main effects	df	Species	AIC	Δ AIC	BIC	logLik
1	SunAlt	5	Ocelot	98458	290	98502	-49224
			Bobcat	37303	46	37341	-18646
2	MoonIlluminatedFrac	5	Ocelot	110042	11874	110086	-55016
			Bobcat	38008	751	38046	-18999
3	TMAX	5	Ocelot	110042	11874	110086	-55016
			Bobcat	37999	741	38037	-18994
4	SunAlt + MoonIlluminatedFrac	6	Ocelot	98460	292	98512	-49224
			Bobcat	37305	48	37351	-18646
5	SunAlt + TMAX	6	Ocelot	98370	202	98422	-49179
			Bobcat	37300	43	37346	-18644
6	SunAlt + MoonIlluminatedFrac + TMAX	7	Ocelot	98372	204	98433	-49179
			Bobcat	37302	45	37356	-18644
7	SunAlt * MoonIlluminatedFrac	7	Ocelot	98461	293	98522	-49224
			Bobcat	37305	48	37358	-18645
8	SunAlt * TMAX	7	Ocelot	98178	10	98239	-49082
			Bobcat	37259	1	37312	-18622
9	SunAlt * MoonIlluminatedFrac * TMAX	11	Ocelot	98168	0	98264	-49073
			Bobcat	37257	0	37341	-18618

Inclusion of variables as additive terms without interaction is indicated by “+” in the Main effects column. Inclusion of an interaction term between independent variables is indicated by “*” in the Main effects column. Models were ranked according to AIC, with the model displaying the lowest AIC per species considered the top model. Bayesian Information Criterion (BIC) and log likelihood (logLik) are included for comparison purposes. All models included individual ID and “Type” (i.e., True Velocity or Predicted Velocity) as random effects. A first-order autoregressive structure was incorporated into all models to account for temporal autocorrelation in the data sets.

**Table 3:** Model coefficients estimates (Value) with t-value and significance for all fixed effects included in Model 9 for ocelots and bobcats.

Species	Fixed Effect	Value	Std.Error	DF	t-value	p-value
Bobcat	(Intercept)	0.067	0.234	15546	0.29	0.7751
	SunAlt	0.021	0.005	15546	4.43	<0.0001*
	MoonIlluminatedFrac	-0.628	0.360	15546	-1.74	0.0810
	TMAX	-0.005	0.003	15546	-2.12	0.0341*
	SunAlt:MoonIlluminatedFrac	-0.013	0.007	15546	-1.81	0.0710
	SunAlt:TMAX	0.000	0.000	15546	-5.40	<0.0001*
	MoonIlluminatedFrac:TMAX	0.007	0.004	15546	1.76	0.0784
	SunAlt:MoonIlluminatedFrac:TMAX	0.000	0.000	15546	1.69	0.0911
Ocelot	(Intercept)	-1.072	0.120	43364	-8.93	<0.0001*
	SunAlt	0.008	0.002	43364	4.54	<0.0001*
	MoonIlluminatedFrac	0.086	0.152	43364	0.56	0.5726
	TMAX	0.006	0.001	43364	6.23	<0.0001*
	SunAlt:MoonIlluminatedFrac	-0.012	0.003	43364	-4.13	<0.0001*
	SunAlt:TMAX	0.000	0.000	43364	-11.65	<0.0001*

Interactions between variables are indicated by “:”. Significant p-values (alpha = 0.05) are indicated by a “*” superscript.

### Discrete time period comparisons

**Fig 3:**
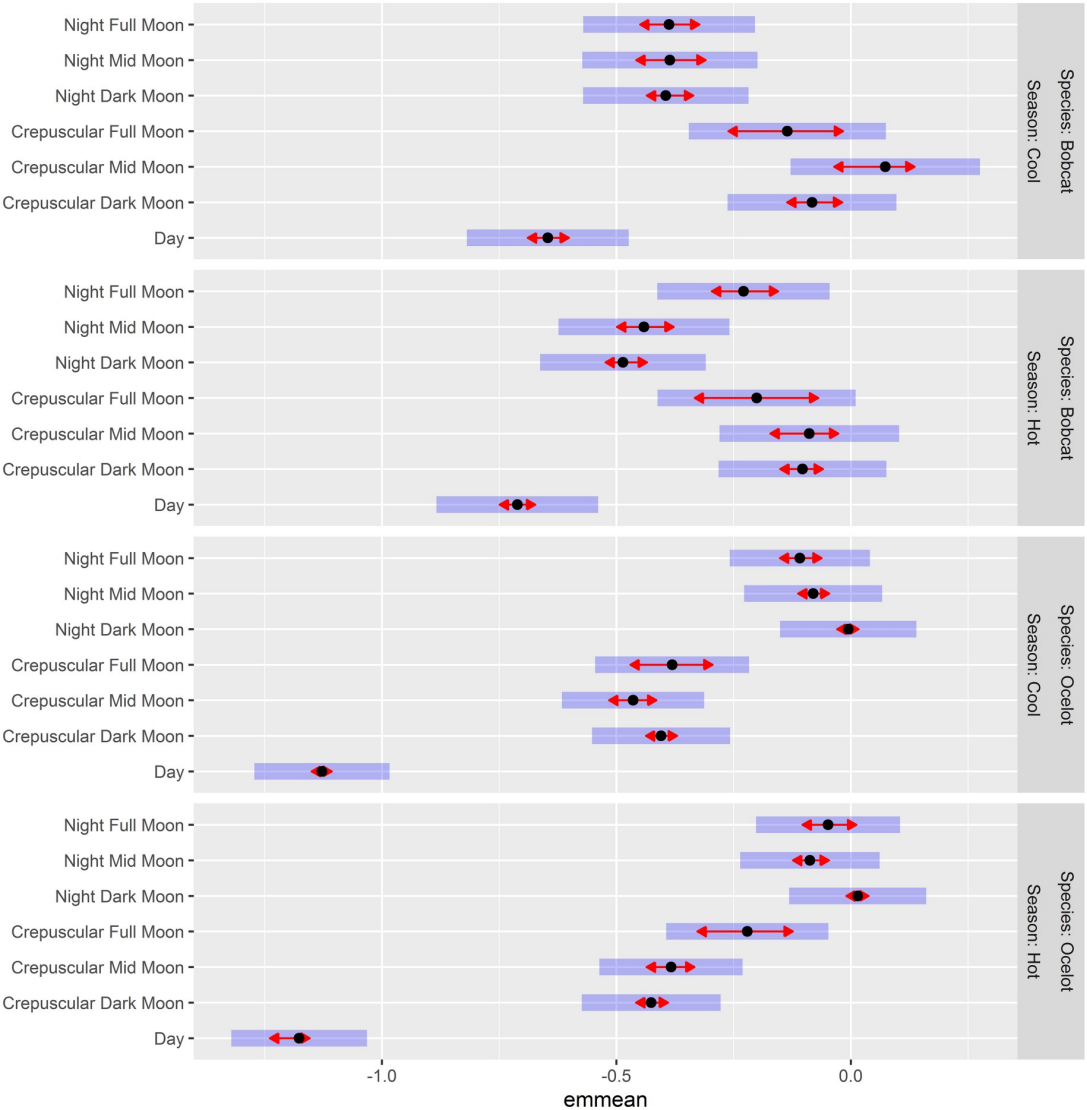
Pairwise comparisons of ocelot and bobcat log Velocity for combinations of season, lunar illumination, and diel period. Estimated marginal means (emmeans) displaying predicted log Velocity of ocelots and bobcats trapped on the East Foundation’s El Sauz Ranch between 2013 and 2017. Estimated marginal means are grouped according to Season and Species, with all combinations of diel period and lunar illumination displayed within each grouping. Estimated marginal means are displayed as black dots, with 95% confidence intervals represented by the purple bars. Red arrows allow statistical comparisons between groups of interest. Non-overlapping arrows are indicative of movement rates that are significantly different (α = 0.05) from each other.

The final model selected for ocelot and bobcat activity comparisons included LunarDiel, Species, and Season as fixed effects with interaction terms. We performed two types of comparisons using estimated marginal means: 1) comparison of activity levels between time periods for a single species, and 2) comparison of ocelot and bobcat activity levels within a specific time period. For bobcats, we observed the following patterns in activity levels: 1) During the hot season, bobcats had higher velocity on full moon nights than on either mid moon (p < 0.01) or dark moon (p < 0.01) nights, 2) During the cool season, activity levels were similar across all lunar illumination levels for both night and crepuscular periods (p > 0.05), 3) During the hot season, crepuscular activity was higher than nighttime activity during dark (p < 0.01) and mid moon periods (p < 0.01), but not during the full moon (p = 1), 4) During both seasons, daytime activity was lower than either nighttime (p < 0.01) or crepuscular activity (p < 0.01), 5) Bobcat daytime activity was slightly higher during the cool season than during the hot season, though the difference did not reach statistical significance (p = 0.22) (Fig 3; S1 Table).

For ocelots, the following patterns were observed: 1) During the cool season, nighttime activity was higher during dark moon periods than during full moon (p = 0.001) or mid moon periods (p = 0.01), 2) During the hot season, nighttime activity was higher during dark moon periods than during mid moon periods (p < 0.01), but was similar to activity levels during full moon periods (p = 0.64), 3) During the cool season, activity levels were similar across all lunar illumination levels for the crepuscular period (p > 0.05), 4) During the hot season, crepuscular activity levels were higher during full moon than dark moon periods (p = 0.001), but not mid moon periods (p = 0.079), 5) For all seasons and lunar illumination levels, activity levels were highest at night and lowest during the day, with intermediate levels occurring during the crepuscular period (p < 0.05), 6) Ocelot daytime activity was higher during the cool season than during the hot season (p = 0.03) (Fig 3; S1 Table).

Comparisons between ocelots and bobcats during specific time periods revealed the following differences: 1) Daytime activity was higher for bobcats than for ocelots during both seasons (p < 0.01), 2) Nighttime activity was higher for ocelots than for bobcats for all season and moon phase combinations except for Hot Season Full Moon Nights (p < 0.05), 3) Crepuscular activity levels were higher for bobcats than for ocelots during Cool Season Dark Moon Crepuscular (p = 0.01), Cool Season Mid Moon Crepuscular (p < 0.01), Hot Season Dark Moon Crepuscular (p = 0.01), and Hot Season Mid Moon Crepuscular (p = 0.02), 5) For all time periods of interest, bobcats and ocelots had similar activity levels only during Hot Season Full Moon Crepuscular (p = 0.88), Cool Season Full Moon Crepuscular (p = 0.07), and Hot Season Full Moon Night (p = 0.13). All other comparisons between ocelot and bobcat activity levels yielded significant differences (Fig 3; S2 Table).

## Discussion

Interspecific competition within carnivore guilds can be particularly intense and can impact the density, distribution, and behavior of carnivore species [68]. Interference competition between ecologically similar species may influence species distribution by relegating subordinate species to marginal habitats [68–71]. Whereas the ocelot is a federally-listed endangered species in the United States, occurring in only two isolated populations in South Texas, the bobcat is the most abundant and widely-distributed native felid in North America [24]. Additionally, the ocelot shows a higher level of body-size overlap with the bobcat than with any other felid that occurs within the ocelot’s range, suggesting that ocelots and bobcats may compete for the same prey items. Potential temporal niche partitioning between sympatric ocelots and bobcats in South Texas is important from a conservation perspective. If bobcats are found to limit the population size or distribution of ocelots in South Texas, bobcat removal may be considered as a management option to increase the sizes of remaining ocelot populations. Additionally, bobcats are widely considered habitat generalists that are able to adapt to human-dominated landscapes [24], whereas ocelots are habitat specialists that are highly dependent on dense cover [13]. Further anthropogenic modification of the South Texas environment may favor an increase in bobcat numbers to the detriment of ocelots.

The overall diel-based activity patterns observed for ocelots are consistent with previous studies [e.g., 19, 44–46] which have reported ocelots to be more active at night than during the day. However, we acknowledge that previous studies of ocelot temporal activity patterns have taken place in a variety of environments which are very different from the South Texas ranchlands where we conducted our research. We found no evidence indicating that ocelot activity peaks during the crepuscular period. Rather, our *post-hoc* emmeans comparisons revealed ocelot activity to be highest at night and lowest during the day, with intermediate activity levels during the crepuscular period.

In contrast, bobcats showed a crepuscular pattern of movement, with estimated marginal mean velocity values typically higher during crepuscular periods than at night, and the lowest activity levels during the day. These results contrast with Rockhill et al. [48] which reported the highest movement rates for bobcats during crepuscular and diurnal periods, with the lowest movement rates occurring on moonless nights. However, the study conducted by Rockhill et al. [48] took place in the Southeastern United States, in an environment very different from the one in which we conducted our research. As such, the differences between our study and other studies of bobcat activity patterns may be due to behavioral plasticity of the bobcat.

Our study found limited support for the hypothesis that bobcats and ocelots show different nocturnal activity patterns due to moon phase. During the hot season, bobcats showed the highest activity levels on Full Moon Nights, whereas ocelots showed the highest activity levels during dark moon nights during both the hot and cool seasons. These differences may point to fundamental differences in the hunting strategies of ocelots and bobcats due to different levels of adaptation to low-light conditions. Banks et al [72] found a strong correlation between the pupil shape of terrestrial species and ecological niche. Species with vertically elongated pupils tended to be nocturnal ambush predators, whereas species with round or subcircular pupils were more likely to be diurnal. The study characterized the ocelot as a nocturnal ambush predator with vertical pupils and the bobcat as a polyphasic ambush predator with subcircular pupils. More research should be done on the eye structures and visual abilities of ocelots and bobcats to determine if the observed differences in temporal activity patterns result from biological differences in nighttime visual acuity. Additionally, anthropogenic light sources may increase total nighttime light availability regardless of lunar illumination levels. If bobcats are more adapted to nocturnal hunting with intermediate to high levels of ambient light, anthropogenic light pollution may give them a competitive advantage over ocelots. The ranchlands where we conducted our research are far from anthropogenic light sources, however, continued urban expansion in South Texas may eventually lead to nighttime light levels that are beneficial to bobcats and detrimental to ocelots. We acknowledge that moon phase and altitude offer crude approximations of nighttime ambient light levels, as light availability may be either amplified or reduced by cloud cover [73]. We initially explored including cloud cover into our estimation of nocturnal lunar illumination, but determined that the cloud data available were insufficient for such estimates.

Our results supported the hypothesis that ocelots reduce diurnal activity during warmer periods of the year. For bobcats, daytime activity levels were slightly lower during the hot season than during the cool season, but this difference did not reach statistical significance (α = 0.05). Although activity levels were lower during the day than during any other time period for ocelots and bobcats, both ocelots and bobcats showed occasional bouts of diurnal activity. Overall, bobcat activity was higher during crepuscular periods than at night, however, during the hot season, nighttime full moon activity was similar to crepuscular full moon activity. These results are somewhat consistent with Schwab et al [42] which reported bobcats to show a crepuscular movement pattern during the winter and a nocturnal pattern in the spring. Although our study area was different from the Mojave desert ecosystem where Schwab et al [42] conducted their research, both areas are characterized by extreme daytime heat (> 33°C) during the summer. Schwab et al [42] reported that bobcats often rested among boulder piles when daytime temperatures became excessive. Bobcats in our study area may be engaged in similar behavior during hot daytime periods.

Males of both species tended to move greater distances than females, yet our study lacked sufficient sample size to include sex as a model parameter. Rather, we accounted for individual variation in movement by including individual ID as a random effect in all models. Prior to conducting our analysis we examined all GPS locations for female ocelots and bobcats to look for denning behavior (i.e., repeated returns to the same local vicinity) but found none. The presence of denning behavior could have confounded our analysis, as denning females are likely to show different activity patterns from non-denning individuals [74].

Our *post-hoc* comparisons found strong evidence for temporal niche partitioning between ocelots and bobcats, with similar activity levels detected only during the following time periods: 1) Cool Season Full Moon Crepuscular, 2) Hot Season Full Moon Crepuscular, and 3) Hot Season Full Moon Night. These time periods are all characterized by intermediate levels of ambient light, and they may indicate times when ocelots and bobcats are similarly active and competing for the same resources. These findings are similar to those reported by Cozzi et al. [75], which found evidence for reduced temporal partitioning among African carnivores during full moon periods.

Our study demonstrates that accelerometer readings can be used to predict movement rates for ocelots and bobcats, even when the accelerometer readings are averaged over 5-minute intervals, and fine scale acceleration patterns are obscured. Tri-axial accelerometer data has been used to characterize animal behaviors of varying complexity, including locomotion, feeding, and patterns related to social interactions [49]. Such classification of complex behaviors requires that accelerometer data be collected at the same time behavioral data are recorded by an observer. Additionally, such studies typically use raw static and dynamic acceleration values in 3 axes to determine animal position and movement. This study lacked observational data on collared ocelots and bobcats, and the accelerometer data collected were reported on only two axes and were averaged over 5 minute time intervals. Nevertheless, we demonstrated that even this coarse-scale accelerometer data can be used to predict movement velocity of free-ranging ocelots and bobcats.

A potential problem with using movement velocity as a measure of activity level is that individuals may be highly active in a local area without moving great distances between consecutive GPS locations. In such cases, a period of high activity may be assigned a low movement velocity value. With properly-functioning accelerometers, large movement distances should always correspond with high accelerometer values, yet not all high accelerometer values are indicative of large movement distances. Our study predicted movement velocity using accelerometer data. We found a strong linear relationship between velocity and Predicted Velocity, though the relationship showed greater variance at higher velocity values. This pattern may be an inherent shortcoming of accelerometer-based movement predictions, as high-velocity movement periods are likely to involve greater variation in corresponding accelerometer values than low-velocity movement periods. Future studies should attempt to characterize more complex movement patterns (e.g., resting, walking, running, pouncing) using original tri-axial accelerometer readings and direct observations of captive individuals. Such a study might find ocelots and bobcats to be engaged in different activities during time periods where overall movement rates are similar.

## Supporting information

S1 Table Estimated marginal means (emmeans) comparisons of log Velocity for ocelots and bobcats for all possible combinations of LunarDiel and Season. The pairwise comparison of interest is indicated by “contrast”. LunarDiel period is reported first, followed by Season. The second time period of interest within the contrast column is subtracted from the first, yielding a positive value in the “estimate” column if first time period had higher values than the second, and a negative value otherwise. Standard error of the mean, degrees of freedom, and t-ratio are provided by the columns “SE”, “df”, and “t.ratio”, respectively. The column “p.value” tests the null hypothesis of no difference in estimated marginal means between the two time periods of interest.
(CSV)

S2 Table Estimated marginal means (emmeans) comparisons of log Velocity between ocelots and bobcats for all possible combinations of LunarDiel and Season. The pairwise comparison of interest is indicated by “contrast”. Ocelot emmeans are subtracted from bobcat emmeans, yielding a positive value in the “estimate” column if bobcat values are higher than those of ocelots, and a negative value otherwise. Standard error of the mean, degrees of freedom, and t-ratio are provided by the columns “SE”, “df”, and “t.ratio”, respectively. The column “p.value” tests the null hypothesis of no difference in estimated marginal means between ocelots and bobcats.
(CSV)
